# Mediodorsal thalamus lesion increases paradoxical sleep in rats

**DOI:** 10.5935/1984-0063.20190155

**Published:** 2021

**Authors:** S N Sriji, Nasreen Akhtar, Hruda Nanda Mallick

**Affiliations:** All India Institute of Medical Sciences, Department of Physiology - New Delhi - Delhi - India.

**Keywords:** Mediodorsal Thalamic Nucleus, Sleep, Ibotenic Acid, Glutamic Acid

## Abstract

**Introduction:**

The mediodorsal thalamic nucleus has extensive connections with prefrontal cortex, which is considered as seat of cognition. It also receives connections from sleep-wakefulness regulating areas in the brainstem and hypothalamus. Decreased volume and degeneration of mediodorsal thalamic nuclei have been reported in schizophrenia and fatal familial insomnia, respectively. In both conditions, the sleep is abnormal.

**Objective:**

To study the role of mediodorsal thalamic nuclei in sleep wakefulness in rats.

**Material and Methods:**

Neurotoxic lesion of mediodorsal thalamic nuclei with ibotenic acid was performed in adult male Wistar rats and sleep wakefulness was recorded. The recordings were taken on 2nd, 7th and 14th days after lesion and compared with the baseline recordings. In order to study the diurnal changes, lesion recordings were of 24h duration. We also performed L-glutamate excitation of mediodorsal thalamic nuclei in another set of animals. After L-glutamate microinjection, sleep wakefulness was recorded for 4h. The recordings were obtained in a digital acquisition system (BSL 4.0 MP 36, Biopac Systems, Inc., USA).

**Results:**

In the present investigation, ibotenic acid lesion of mediodorsal thalamic nuclei reduced the wakefulness and increased paradoxical sleep, which contradicts the reports from earlier lesion studies in cats. Glutamate excitation of mediodorsal thalamic nuclei produced prolonged wakefulness.

**Discussion:**

The results suggest that the mediodorsal thalamic nuclei augments arousal in the ascending reticular wake promoting pathways in contrast to the earlier reports that mediodorsal thalamic nucleus is involved in generation of slow wave sleep. The present study adds another evidence for the role of thalamus in sleep-wake regulation.

## INTRODUCTION

One of the higher order thalamic nuclei, the mediodorsal thalamic (MD) nuclei is extensively connected with the prefrontal cortex and involved in higher cognitive functions^1,2^. The MD also receives broad spectrum of afferent projections from the areas involved in regulation of sleep wake cycle, which include the tegmental areas, brainstem reticular nucleus, posterior hypothalamus, raphe nuclei and preoptic anterior hypothalamus^3,4,5^. Two clinical conditions, fatal familial insomnia (FFI) and schizophrenia share some common features like: inability to sleep, reduction in sleep spindles and K complexes and disruption of the cyclic sleep organization^6,7^. The underlying pathophysiology in both the cases involve severe degeneration of the MD nuclei^8,9^. These are due to large ischemic lesions that affects large area in MD.

Thalamus by virtue of its extensive network connections with cortical as well as subcortical structures plays a dual role in sleep wakefulness. The nonspecific thalamic nuclei are part of the general arousal system and the thalamocortical circuitry that orchestrates the generation of spindles and delta sleep^10,11^. But the specific role of MD in sleep wakefulness regulation is not looked into. In the present study, the role of the MD in sleep wakefulness was studied after lesion and stimulation of the MD in rats.

## MATERIAL AND METHODS

### Animals

The experiment was conducted in adult male Wistar rats (250-300g bwt). The animals were housed with controlled light dark cycle (14:10 h) and at an ambient temperature of 25 ± 1°C. Food and water were provided *ad libitum*. All experiments were performed in accordance with the guidance of Institutional Ethics Committee, All India Institute of Medical Sciences, New Delhi, India & Committee for the purpose of control and supervision of experiments on animals (862/IAEC/15). All efforts were made to minimize the number of animals used and their suffering

### Surgery

The animals were anesthetized with sodium pentobarbital anesthesia (50mg/kg body weight, i.p.) and fixed on a stereotaxic apparatus (Narishige Scientific Instrument Lab., Japan) for implantation of electrodes and cannula. Electroencephalogram (EEG), electromyogram (EMG) and electrooculogram (EOG) electrodes were chronically implanted on the skull and connected to an IC socket fixed on the skull, for the assessment of sleep-wakefulness (S-W), as described earlier^12^. A bilateral guide cannula made from 24G stainless steel with indwelling styli was implanted 2mm above the MD according to the coordinates (AP: -3.00mm, ML: ±1mm, DV: + 5.6mm) from Paxinos and Watson^13^ for drug delivery. Animals were given 1 week for recovery from surgical trauma during which antibiotic (gentamicin sulphate, 2mg/kg bwt IM) and analgesic (meloxicam 1 mg/kg bwt IM) injections were performed for 3 days respectively.

### Lesion of MD

Ibotenic acid (Wako Pure Chemical Industries Ltd., Japan) was used to produce lesion in seven animals. In lesion experiments, the recordings of sleep wakefulness were taken for 24h. After acquisition of the baseline data (8:00-8:00h) on three alternate days, the animals were anesthetized and ibotenic acid microinjection was performed by an injector cannula inserted through the guided cannula. Ibotenic acid (5µg/µl) was injected bilaterally (200nl) using a 1µl Hamiton syringe connected to the stereotaxic injector pump (Pump 11 Elite Nanomite, Harvard apparatus) at a rate of 9.99nl/min. The injection on each side took 20min and the injector remained for another 5min above the site of injection. The interval between bilateral injections was 10-15min. The post lesion recordings were taken on 2nd (D2), 7th (D7) and 14th (D14) days. Friedman test was used to test the statistical significance between the baseline and post lesion values.

### Microinjection of L-glutamate/saline (n=12)

Microinjection of L-glutamate (Sigma Chemicals Co., USA)/saline was performed in unanesthetized animals. Six animals received L-glutamate microinjection (60ng/200nl saline) and six animals received sterile saline microinjection (200nl) microinjection at MD in six rats. In all the animals after surgical recovery, three baseline recordings were taken for 6h (10:00-16:15 h). These baseline recordings served as the time matched control values. During baseline recordings, at 12:00h, the animals were handled and the microinjection procedure was mimicked without any injection in order to reduce the effects produced by handling and insertion of injector into the brain during drug injection. On the day of microinjection, after 2h of baseline recording, the animals received bilateral microinjection of 0.2µl L-glutamate or normal saline. No animal received more than one injection. The post injection recording was continued for 4h. Pre injection (10:00-12:00h) and post injection (12:15-16:15h) values of each parameter were compared between time matched control, L-glutamate injected group and vehicle injected group using Mann Whitney U test. The duration and frequency of episodes of sleep wakefulness were calculated in the entire 4h post injection recording time. The values of the three groups were compared using Kruskal Wallis test.

### Recording of Sleep Wake cycle and animal euthanasia

After recovery period, the animals were exposed to the recording chamber and connecting cables for 48h for habituation prior to the experiments. In animals receiving L-glutamate or saline microinjections, the styli of the guide cannula were removed and kept back in place around 12PM during this time. Sleep wakefulness was recorded by a digital polysomnography (BIOPAC BSL 4.0 M36 system). The sampling rate for EEG, EOG and EMG were kept 125, 125 and 500Hz respectively. The recordings were stored for offline analysis. After the experiments, the animals were euthanized by transcardial perfusion with 4% paraformaldehyde and the brains were taken for histological confirmation of site of injections.

### Histological confirmation of site of lesion and microinjection

Histological confirmation of lesion site: Coronal brain slices of 16µ thickness were stained with cresyl violet for verification of the lesion. Sections were rinsed in distilled water for 1min and stained with cresyl violet stain for 1min. The excess stain was washed out with quick rinse in distilled water. Tissues were then treated with 70% and 95% alcohol for 1min each and in alcohol chloroform mixture for 1min. Sections were then dipped in N butyl alcohol (1min), cleared in xylene and mounted. The neural damage was confirmed by the reactive gliosis and its increase in number.

Confirmation of site of microinjection: The coronal brain sections of 16µ thickness were stained with hematoxylin and eosin stain for identification of the site of injection. The cut sections were washed in distilled water for 1min. The sections were then stained with hematoxylin (1min) and washed under running tap water for 1min. They were then dipped in acid alcohol for 30sec then again washed for 1min under running tap water. Sections were stained with eosin stain for 2min and dehydrated with different grades of alcohol (70%, 90% and 100%) and acetone. The stained sections were cleared in xylene and mounted in DPX for microscopic examination.

### Data Analysis

The data from animals where the injection was confirmed within MD were taken for further analysis. After obtaining the recordings, visual scoring was performed to identify different stages of sleep-wakefulness. The sleep-wake stages were classified into active wake (W1), quiet wake (W2), light slow wave sleep (S1), deep slow wave sleep (S2) and paradoxical sleep (PS)^14^. The percentage values for sleep total sleep time (TST), total wake time (TWT), total slow wave sleep (SWS1 & SWS2), total paradoxical sleep (PS), frequency and duration of wake, SWS and PS were analyzed from the visually scored data.

## RESULTS

### Lesion of MD

**Figure 1:**
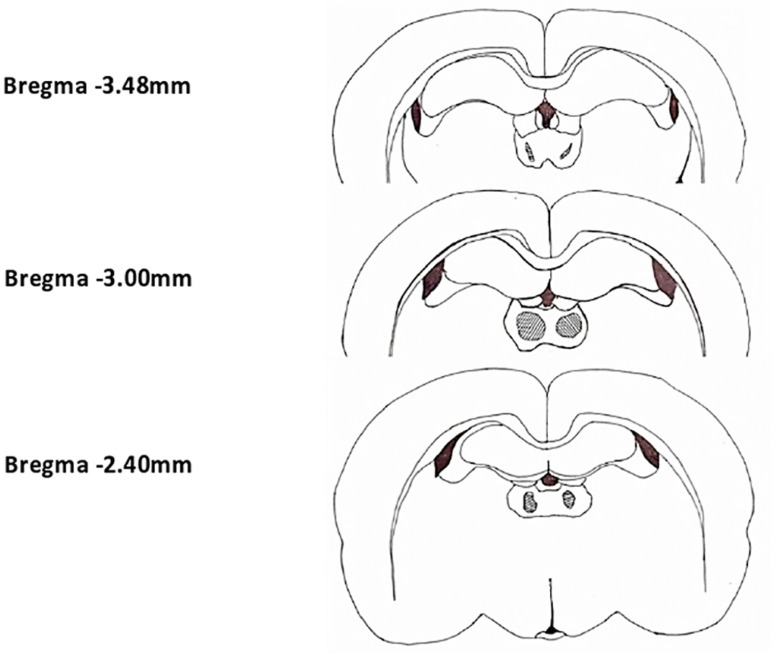
Schematic diagram showing extend of ibotenic acid lesion in MD with respect to bregma drawn according to Paxinos and Watson atlas. The black/grey shaded area shows the area where neuronal damage was prominent in histological examination.

**Figure 3:**
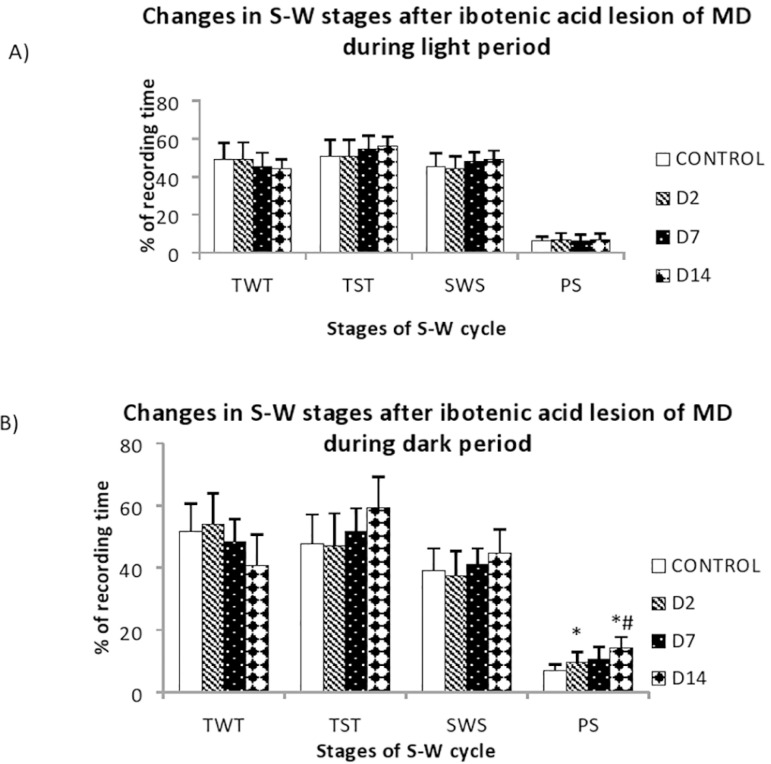
**A.** Bar diagram shows pre (control) and post lesion (D2, D7 and D14) percentage values of different S-W parameters in rats with ibotenic acid lesion at MD during dark period; **B.** Bar diagram shows pre (control) and post lesion (after 2, 7 and 14 days; D2, D7 and D14) values of the frequency of different PS in rats with ibotenic acid lesion at MD. Data expressed in mean ± SD. Asterisk (*) represents significant difference when compared with the control. Hashtag (#) represents significant difference when compared with D2 (*p≤0.05, #*p*≤0.05).

**Figure 4:**
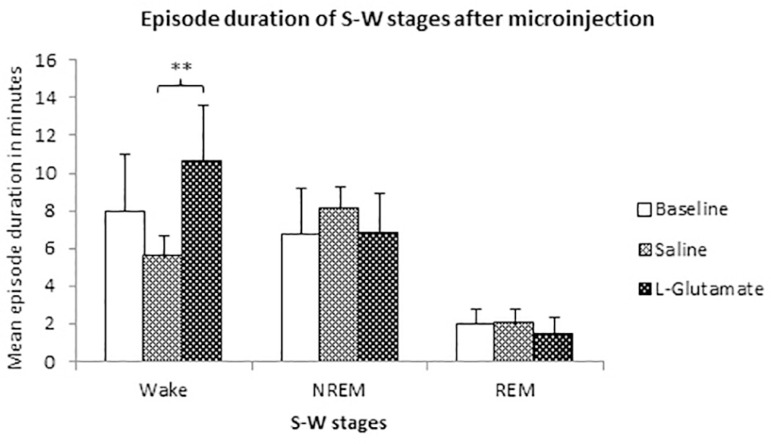
Bar diagram shows pre (control) and post lesion (D2, D7 and D14) values of the frequency of PS in rats with ibotenic acid lesion at MD (Data expressed in mean± SD) (*p≤0.05).

Ibotenic acid produced restricted lesion within the MD. The lesion extended from -2.40mm to -3.48mm (Figure 1) in anteroposterior direction. After ibotenic acid lesion, significant changes were observed in the percentage of PS during dark period, with a significant increase in the frequency of PS episodes. The percentage of PS showed a progressive increase during the dark period, from 6.89 ± 2.04% to 14.36 ± 3.38% (Figure 3B). This increase was significant (*p*<0.05) in the D2 (9.65 ± 3.3%) and D14 (14.36 ± 3.38%). The frequency of REM episodes increased significantly from 38.88 ± 5.81 in control to 54 ± 7.6 D14 (*p*=0.043) (Figure 4). The other parameters also showed variations after lesion without statistical significance. Though statistically not significant, there was remarkable reduction in the TWT after lesion with a corresponding increase in the TST, in a progressive manner from control to post lesion 14th day (Figure 3B). The reduction in TWT was more prominent during the dark period.

The changes observed after L-glutamate injection was significantly different from the changes observed after saline microinjection. Compared to saline injection, L-glutamate injection produced significant increase in TWT and decrease in TST and SWS in the first and third post injection hours. The TWT also showed a significant difference between vehicle injected and glutamate injected group in the post injection third hour (Table 1).

**Table 1:** Post injection values of different S-W parameters in L-glutamate injected and saline injected rats with their time matched control values

Time		12:15 - 13:15 h	13:15 - 14:15 h	14:15 -15:15 h	15:15 -16:15 h
**Stages**					
**PS**	Time matched control	5.32±4.39	7.14±5.92	4.94±2.99	7.67±6.32
	L-Glutamate	2.14±4.789	8.21±5.69	11.25±10.07	9.10± 5.80.
	Time matched control	6.63±4.52	11.21±5.60	9.82±3.0	6.18± 8.03
	Saline	4.09±4.54	7.50±7.49	7.43±5.05	10.83±5.48
**TWT**	Time matched control	27.32±17.06	18.33± 7.77	41.25±16.90	28.69±17.5
	L-Glutamate	82.32±20.14***	22.08± 9.31	40.35±15.83	27.08±23.59
	Time matched control	32.11±20.13	28.33±17.15	23.29±16.53	15.27±24.59
	Saline	54.09±19.50	18.81± 16.26	20.55±10.76	22.2±17.85
**TST**	Time matched control	58.80± 21.23	67.79± 22.74	56.84±13.98	60.47±13.33
	L-Glutamate	17.26±10.56 ***	80.77± 8.93	71.96±19.69	83.03±19.47
	Time matched control	71.14±21.90	79.54± 14.42	85.17± 11.61	34.65±42.08
	Saline	45.83± 19.55	76.66± 14.52	79.72±11.12	77.77±17.85
**SWS**	Time matched control	53.48± 20.37	60.65± 20.86	51.90±12.86	52.79±12.72.
	L-Glutamate	15.11± 9.92***	72.55± 7.19	60.71± 12.49	73.92± 17.41 *
	Time matched control	64.51±18.43	68.33± 12.85	75.34±9.77	28.47± 34.36
	Saline	41.73±15.62	69.16± 11.39	72.29±9.93	66.94±17.28

Table shows percentage values of 4h S-W parameters (mean±SD) in 1h bin in L-glutamate injected and saline injected group with their time matched control values. PS-paradoxical sleep, TWT-total wake time, TST-total sleep time, SWS-total slow wave sleep. Asterisk (*) represents significant difference when compared with the control.

* p≤0.05,

*** p<0.001.

**Figure 5:**
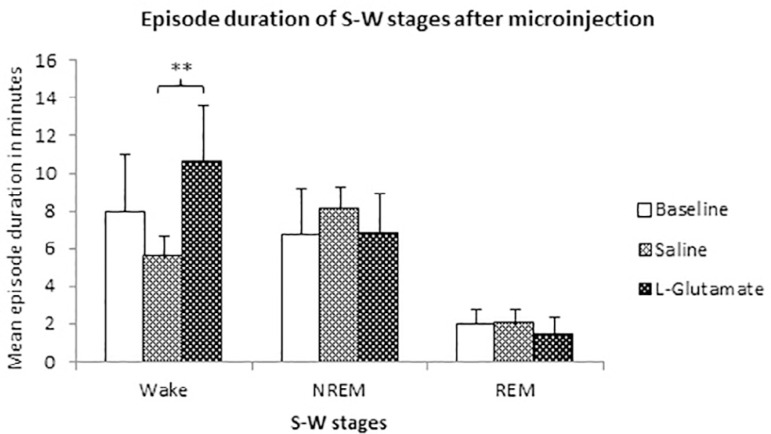
Bar diagram shows average duration per episode of S-W stages in control, vehicle injected and glutamate injected rats. Significant increase in duration of W was observed in glutamate injected group than vehicle injected group. Data is expressed as mean ± SD. Asterisk (*) represents significant difference when compared with the control (**p<0.01).

The frequency of S-W stages did not show any difference in the post injection period. However, the duration of W was significantly increased after L-glutamate microinjection compared to saline injected group (10.56 ± 3.01, *p*=0.009) (Figure 5).

### Microinjection of L-glutamate/saline (n=12)

**Figure 2:**
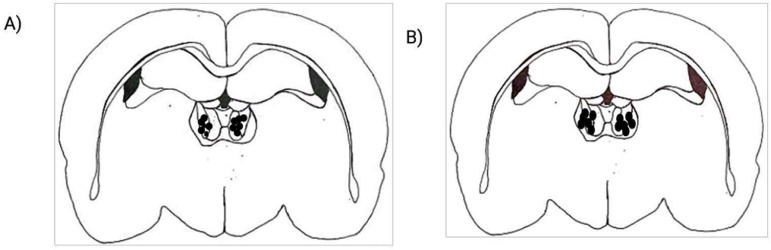
Shows the different sites of microinjections at MD. **A.** Sites of microinjection of glutamate (n=6); **B.** Sites of microinjection of saline (n=6). The filled circles inside the brain sections represent the central points of injection in animals of each group, where the tip of the injector was found to be at MD. Scale bar: 1mm.

In all the rats, the sites and extent of injections were confirmed in MD after histological examination (Figure 2). The pre-injection values L-glutamate injected and saline injected group did not show any significant difference in intra group and intergroup comparisons. After L-glutamate microinjection, there was a significant increase in TWT in the first hour following microinjection, compared to the time matched baseline value (Table 1). The percentage has increased from 27.32 ± 17.06% to 82.32 ± 20.14% (*p*=0.001). On the other hand, the sleep components SWS and TST showed a significant reduction in their percentage values following microinjection in the first hour after microinjection. The total SWS reduced significantly in the first hour after microinjection. The value was reduced from 53.48 ± 20.37% to 15.11 ± 9.9% (*p*=0.001). The TST was reduced from 58.80 ± 21.23% to 17.26 ± 10.56% (*p*=0.001). In the fourth hour after microinjection, the total SWS has increased significantly than the time matched control value, from 52.79 ± 12.72% to 73.92 ± 17.41% (*p*=0.038).

## DISCUSSION

Ibotenic acid lesion of MD decreased the percentage of wakefulness with an increase in light slow wave sleep. There percentage of PS sleep was increased in the expense of wakefulness during dark period following lesion. The TST did not show any changes after lesion. The result obtained in the present study is in marked contrary to the earlier lesion studies of MD on sleep wakefulness. Ibotenic acid as well as electric lesions of MD produced a significant reduction of SWS and PS sleep in cats^15,16^ where the lesion was specific to the intermediate segment of MD.

Lesion was comparatively small to produce large significant changes in the present study. In ibotenic acid lesion, the fibers of passage are spared. This may be the reason, why the slow waves or spindles were not affected after lesion as observed in the earlier studies in cats. The extent of lesion produce various effects even the area involved are same^17^.

The cholinergic neurons fire at wakefulness and PS while the monoaminergic, histaminergic and orexinergic neurons fire only during wake and cease firing during PS^18^. In other words, the cholinergic neurons promote PS and the monoaminergic neurons suppress PS. Direct excitation of brainstem reticular neurons or inhibition of GABAergic inhibition on brainstem RF by the PPT/LDT cholinergic neurons initiates PS. The PS off neurons of raphe nucleus and the locus coeruleus inhibit the PPT/LDT neurons to turn off the PS. Thus the brainstem contains the neural machinery for PS while the forebrain mechanisms modulate it^19^. Since the PS is preserved and wakefulness is reduced, probably the monoaminergic system would have affected after lesion of MD in the present study.MD receives moderate amount of projections from the raphe nuclei (median and dorsal) and the locus coeruleus^20,21^. The lesion would have caused a synaptic dysfunction between LC, raphe nucleus with MD and thereby an imbalance of cholinergic and monoaminergic systems. In general terms, it can be assumed that the balance between the monoaminergic and cholinergic neurons is maintained at some level in MD. It can also be assumed that, there would be dissociation between the PS active and wake active neurons of the brainstem, which passes through MD.

There is suggestive evidence that cholinergic systems may be overactive in schizophrenia^22^. Since MD dysfunction in schizophrenia is pathological feature; the increased PS percentage may be read in line with this. However, in general, patients with acute, short-term illness show reduced REM sleep and chronic institutionalized patients have actually shown increase in percentage of REM sleep^23^. A decrease in REM latency and increase in total REM sleep and REM density and REM sleep alterations are major polysomnographic features of depression^24^.

Similarly, in FFI, the clinical feature, insomnia may not be due to the underlying MD degeneration. There are some FFI reports with selective degeneration of MD nuclei but without insomnia. An extensive thalamic involvement rather than a selective nuclear degeneration account for the dysfunction of circadian mechanisms underlying insomnia in FFI^25^.

In contrary stimulation of the MD with excitatory neurotransmitter, glutamate produced significant increase in wakefulness in rats. This increase in wakefulness was largely due to an increase in the duration of episodes of wakefulness, rather than an increase in the number of wakeful episodes. Glutamate can induce SWS, wakefulness or REM depending on the neural circuit where it acts upon^26,27^. The MD-PFC reciprocal connections are glutamatergic and excitatory^1,28^. Therefore, the arousal period enhanced by L-glutamate injection is due to excitation of MD neurons. In a similar experiment, in anesthetized rats, electrical stimulation of MD produced excitatory effects in the prefrontal cortex^29^. On the other hand, GABA agonist muscimol injection in the MD suppressed the cortical metabolic activity. Suppression of MD-cortical neurotransmission produced attention disturbances and frontal cortical dysfunction^30^.

Involvement of MD in higher cognitive functions such as memory, attention and learning are well established^31^ all of which requires elevated wakefulness.

Projections from reticular formation ascend to the forebrain where they stimulate cortical activation via a dorsal relay in the thalamus and a ventral relay through the hypothalamus and basal forebrain^18^. The dorsal pathway of ascending reticular activating (ARAS) system has a cephalic thalamic ending^32^ this thalamic component of ARAS upon high frequency simulation was able to produce EEG desynchronization and might be taking part in mediating the arousal^33^.

But in another set of experiments, true sleep appeared only after electrical stimulation of the medial thalamic region^34^. The thalamic components of the ARAS system were later identified as the midline and intralaminar group of nuclei^20^. ARAS exerts a major influence on wakefulness and sleep through these pathways originating from the brainstem reticular formation and terminating within the midline and intralaminar thalamic nuclei. MD is part of these diffuse thalamic nonspecific nuclei. The arousal effect of ARAS is transferred to the cortex by the diffuse thalamocortical projections.

All divisions of MD receive common inputs from several brain areas including the reticular nucleus of thalamus, the mesopontine reticular formation, PPT, LDT, LC and the dorsal and median raphe nuclei of the brainstem which are key components of the arousal system^35,36^. MD also receives substantial amount of afferents from basal forebrain (BF) and preoptic anterior hypothalamus^5^. Projections from mesopontine cholinergic neurons to thalamus appear to form a component of involved in brain-state control, and are thought to be involved in regulating states of arousal, attention and the sleep-wake cycle^4^.

From the increased duration of wake episode than its number of occurrence, it can be hypothesized that the MD takes part in maintaining the arousal state rather than initiating it. This is in line with the hypothesis that thalamus end of the reticular formation mediates the arousal. The MD may act as a hub for the wake regulating basal forebrain and brainstem afferents making a final common pathway to cortical arousal. After the prolonged wakefulness, the L-glutamate injected rats entered to the normal sleep wake pattern after 1h. But the slow wave sleep has increased significantly in the fourth hour after microinjection. This increase in slow wave sleep can be understood as a result homeostatic increase in sleep drive due to the extended prior wakefulness^37^.

In a recent study, the stimulation of paraventricular thalamic nucleus (PVT) produced wakefulness^38^. MD and PVT are part of paramedian thalamus, which in general are considered to be involved in circuitry controlling wakefulness. The hypersomnia following paramedian thalamic stroke is thought to be due to the inability of the arousal network to maintain wakefulness^17^.

## CONCLUSION

The results of the present study show wake promoting role of MD neurons. MD is strategically located to enhance the ongoing ascending reticular wake promoting information, disruption of which may result in the imbalance of wake and sleep.
